# Adult male involvement in maternity care in Enugu State, Nigeria: A cross-sectional study

**DOI:** 10.18332/ejm/112258

**Published:** 2019-09-23

**Authors:** Chisom J. Mbadugha, Chinenye J. Anetekhai, Adaobi L. Obiekwu, Ijeoma Okonkwo, Justin A. Ingwu

**Affiliations:** 1 Department of Nursing Sciences, Faculty of Health Sciences and Technology, University of Nigeria, Enugu, Nigeria; 2 National Orthopaedic Hospital, Enugu Nigeria; 3 Federal Neuropsychiatric Hospital, Enugu, Nigeria

**Keywords:** male partners, obstetric care, maternity care, male involvement

## Abstract

**INTRODUCTION:**

Men are the key decision makers in the family and play a crucial role in the reproductive health of partners, in Nigeria. This study assessed adult male involvement in maternity care in Enugu south local government area, Enugu State, Nigeria.

**METHODS:**

This community-based study was conducted using a cross-sectional survey design. A total of 145 respondents were selected through multi-stage sampling and data were collected using a structured questionnaire developed by the researchers. Data generated were statistically analyzed based on the research objectives using descriptive statistics.

**RESULTS:**

Major findings revealed that the respondents had moderate knowledge on the expected role of males in maternity care with the majority, assessed using a 4-point Likert scale, having a moderate (2.99) level of involvement in maternity care. Lack of facilities that encourage male participation in maternity care, work schedule of the male partner, and lack of knowledge on the role of the male partners during maternity care were identified as major barriers to male involvement in maternity care with means of 3.80, 3.58 and 3.48, respectively.

**CONCLUSIONS:**

Involvement in maternity care among the respondents in this study was moderate. However, men may be restricted by some cultural beliefs such as maternity care being regarded as exclusively a woman’s matter. Thus, men should be educated on the importance of their role as partners in maternity care and on the need to participate actively, regardless of existing cultural norms. Hospitals should also promote policies that encourage male presence during birth and delivery rooms need to be designed to allow bonding of partners during birth.

## INTRODUCTION

Maternal mortality is widespread globally. The World Health Organization reported that 289000 women die from pregnancy and childbirth complications around the world every year, 800 women die daily and 99% of these deaths occur in developing countries^1^. Findings from a local study also found the average maternal mortality ratio in Southern Nigeria to be between 454 and 772 per 100000 births^2^. Although this value is lower than the maternal mortality ratio in Northern Nigeria (2420 per 100000 births), it still falls short of the sustainable development goal targeted at reducing maternal mortality ratio to less than 70 per 100000 births^3^. Hence, there is a need for viable options for improving maternal outcomes. A United Nations report, from an international conference on population and development^4^, defined male involvement in maternity care as ‘a process of change in the social and behavioural domains required of men to play a critical role in reproductive health care, aimed at ensuring the wellbeing of women and their children’. A systematic review of the impact of male involvement in the maternal health outcome of women in developing countries provided three broad categories indicating male involvement: 1) Active participation in maternal care services such as support of the spouse by the husband during pregnancy, childbirth or postpartum, 2) Financial support provided for pregnancy-related and childbirth expenses, and 3) Shared decision making powers with wife on maternal health issues^5^. However, male involvement in this study refers to active participation of men during pregnancy and childbirth. Involvement will be assessed in terms of providing assistance, accompanying to hospital, providing support, and birth preparation (transportation, blood donation and place of delivery).

In patriarchal societies, male partners generally do not accompany their partners to antenatal or postnatal care services and are not expected to be present during the birth of their children^6-7^. Lack of information regarding maternal care services is noted to be a significant factor that impedes male active participation, hence the need for exhaustive education and radical awareness campaigns^8^. Studies have shown that providing men with comprehensive information relating to maternal health issues and services will enhance active participation in maternal care^5^, promote birth preparedness and complication readiness, improve maternal mental health, improve use of maternal health services, and promote maternal and foetal health outcomes^9-12^.

Male involvement is one of the driving forces of maternal mortality reduction. In view of the essentiality of their participation, previous literature has recommended active inclusion and shared responsibility of male partners in maternal health as a measure to improve maternal outcomes^1^. However, there is still a wide gap between male involvement policies and the actual involvement in pregnancy and birth^9^. Furthermore, there is a growing debate among policymakers and researchers on the role of men in maternity care, which is a significant challenge in Nigeria. Despite the fact that men are important in maternal and child health, they have neither played a significant role in pregnancy and childbirth nor a role in reproductive health initiatives. Available evidence^13^ shows that a woman’s ability to seek and use health care services is determined by the household head, which is usually the husband. Also, it is a predominant fact that most Nigerian men are reluctant to participate in the care of their partners during pregnancy and childbirth. This could be a result of cultural beliefs that issues surrounding pregnancy and labour are exclusively women matters.

Anecdotal surveys further show that men, especially of the Igbo population, feel it is out of place to accompany or participate in maternal care services such as pregnancy, childbirth, family planning and immunization. As practising midwives, the researchers entertained complaints by mothers of the challenges they face concerning their reproductive health, especially the lack of support and participation by the male partners during pregnancy, labour, and postpartum. These complaints were validated as most women came alone for antenatal care, while during labour they were dropped off at the ward by their male partners to go through the process of delivery alone. Furthermore, those who accompany their partners to the hospital for antenatal services usually drop them off or wait for them outside without participating in the antenatal consultations and care.

Moreover, there is a paucity of data from the Nigerian community on male involvement in maternity care. Based on the foregoing, the researchers set out to undertake a study to determine the level of knowledge of male partners of their expected role in maternity care, the extent of involvement in maternity care, as well as barriers to involvement in maternity care among adult males in Enugu south local government area, Enugu State, Nigeria.

## METHODS

### Study design – sample and sampling technique

A community-based cross-sectional survey was undertaken for a period of 7 weeks to assess adult male involvement in maternity care in Enugu south local government area, Enugu State, Nigeria. A four-stage sampling technique was used. The first stage involved simple random sampling to select households from the five communities within Enugu south local government area namely, Achara, Amaechi, Obeagu, Ugwuaji, and Uwani. Achara was selected using the lucky-dip method. In the second stage, the number of residential houses in Achara was determined, and 949 were identified (town planning unit of Enugu south local government area, 2017). A sample frame was developed using the primary health care numbering system, and systematic sampling was used to determine the number and houses to be selected. In the third stage, simple random sampling was used to select the first house to be visited. The third household from the entrance into the community was selected; subsequently every 6th house was selected. Finally, purposive sampling was used in each residential building visited to select the respondents. Out of 150 selected households, 5 declined to participate in the study yielding a total sample size of 145 men.

### Inclusion criteria

Men aged ≥20 years, married or cohabiting with a woman who was pregnant or had a baby that was <2 years old at the time of this study and were willing to participate and available during the study period were eligible to participate in the study.

### Data collection instrument

A structured questionnaire developed by the researchers based on previous literature was used for data collection. The questionnaire comprised 27 questions and four sections: sociodemographic characteristics, knowledge of expected roles of men in maternity care with 7 items, their level of involvement with 8 items, and barriers to male involvement in maternity care with 7 items. Participants’ responses on each variable were assessed using a rating scale. Items on knowledge were assessed using a 3-point scale, ranging from 1 (incorrect knowledge) to 3 (correct knowledge). Items on extent and barriers to male involvement were assessed using a 4-point Likert scale, with 1 corresponding to very low extent, 2 low extent, 3 moderate extent and 4 high extent, representing rarely, sometimes, often and always, respectively. The questionnaire items were in English. Meanwhile, the instrument was given to 3 experts in the field of maternal and child health nursing who evaluated its validity. Their judgement was used to modify the tool before using the instrument for field testing. Content validity was calculated using a content validity index (CVI). Experts rated each item on a 4-point scale of relevance (1=not relevant, 2=somewhat relevant, 3=quite relevant, 4=highly relevant). For each item, the cumulative content validity index (I-CVI) was computed as the number of raters giving a rating of 3 or 4, divided by the number of experts. All the experts gave either a value of three or four for all the items giving an I-CVI that was acceptable. This is in line with Lynn^14^ who posited that when there are 5 or fewer experts, the I-CVI must be 1.0 (i.e. all experts must agree that the item is content valid). The instrument was pilot tested. Copies of the questionnaire were administered to 20 male partners in Abakpa Nike community (Enugu east local government area) within a period of 2 weeks. The scores were analyzed using the test-retest method and a reliability coefficient of 0.81 was obtained. The general convention in research, prescribed by Nunnally and Bernstein^15^, posits that reliability values should be ≥0.70. On this basis, the questionnaire was considered reliable.

### Procedure for data collection

Three research assistants who were midwives with a bachelor’s degree and had collected data for other studies were recruited to help with data collection. After thorough explanation of the research purpose and ethics, the research assistants were trained on how to elicit responses from the questionnaire items. Prior visits were made to each of the streets by the research team to introduce the members of the research team, establish rapport with the ward councillor of the community and explain the aims of the study and research protocol. The research team was able to communicate fluently in English and in the local dialect (Igbo). Male partners who met the inclusion criteria were selected. Any building for which information was not ascertained was discarded, and the next house visited in its place. Literate respondents filled the questionnaire independently while the illiterate ones were assisted with the content reading and interpretation in the local dialect. Data collection was done in the evenings and weekends to provide opportunity for the researchers to meet the male partners at home.

### Ethical considerations

Ethical clearance was obtained from the institutional review board, University of Nigeria Teaching Hospital, Ituku-Ozalla and the administrative permit from the Chairman, Enugu south local government area, Enugu State. Respondents were recruited after informed consent was obtained orally. They were assured of anonymity and confidentiality of their data and their freedom to withdraw anytime without any consequences.

### Data analysis

Data collected were sorted out and analyzed with the aid of the IBM statistical package for social sciences (IBM SPSS) software Version 23. Data analysis was done using descriptive statistics (frequencies and percentages for categorical variables; means and standard deviations for numerical variables). Items on knowledge were assessed based on a decision rule that 0–49% represents poor knowledge, 50–69% moderate and ≥70% good knowledge. The questionnaire items on level of involvement and barriers to male involvement in maternity care were rated using a 4-point Likert scale with a mean cut-off of 2.5. Level of involvement was assessed based on: <2.5 low, 2.5–3.25 moderate, and >3.25 high level of involvement.

## RESULTS

The sociodemographic characteristics of the respondents revealed a mean age of 38.34 years (SD=8.93). Of the 145 of respondents, 110 (75.9%) were married, 88 (60.7%) had tertiary level education, 141 (97.2%) were Christians, and 53 (36.6%) were civil servants (Table 1).

**Table 1:** Sociodemographic characteristics of respondents (n=145)

*Variable*	*Categories*	*Frequency*	*Percentage*
**Age groups** (years)	20–29	30	20.7
(Mean=38.34, SD=8.93)	30–39	56	38.6
	40–49	37	25.5
	50–59	21	14.5
	≥60	1	0.7
**Marital status**	Married	110	75.9
	Separated	18	12.4
	Divorced	5	3.4
	Cohabitation	12	8.3
**Educational level**	Tertiary	88	60.7
	Secondary	40	27.6
	Primary	2	1.4
	No formal schooling	15	10.3
**Religion**	Christianity	141	97.2
	Atheist	1	0.7
	Traditional	3	2.1
**Occupation**	Civil servant	53	36.6
	Business man	50	34.5
	Trader	16	11.0
	Artisan	17	11.7
	Unemployed	9	6.2

An assessment of knowledge of the expected role of male partners in maternity care is shown in Table 2. The result shows that the majority of the respondents were not so sure about their expected role in maternity care in all the items. However, the three most correct items identified by the respondents regarding their expected role in maternity care were: helping to take care of the other children 62 (42.8%); giving emotional support during pregnancy 58 (40%); and reminding the partner of her medications, antenatal visits and other examinations 57 (39%). Overall, the respondents demonstrated moderate knowledge of their expected role during maternity care.

**Table 2:** Men’s knowledge* of expected role during maternity care, Nigeria (n=145)

*Variables*	*n (%) Correct*	*n (%) Not so sure*	*n (%) Incorrect*
1. Going for antenatal visits with their partners	47 (32.4)	89 (61.4)	9 (6.2)
2. Helping out with the house chores	46 (31.7)	96 (66.2)	3 (2.1)
3. Taking a walk	37 (25.5)	97 (66.9)	11 (7.6)
4. Cooking for the spouse	37 (25.5)	59 (40.7)	48 (33.1)
5. Taking note of the expected date of delivery to make preparations	47 (32.4)	56 (38.6)	42 (29.0)
6. Helping out in taking care of the other children	62 (42.8)	82 (56.6)	1 (0.7)
7. Giving emotional support throughout the pregnancy	58 (40)	85 (58.6)	2 (1.4)
8. Reminding her of medication and antenatal visits	57 (39)	86 (59.3)	2 (1.4)

* Knowledge of expected role: ≥70% good; 50–69% moderate; 0–49% poor.

**Table 3:** Extent of male involvement* in maternity care, Nigeria (n=145)

*Variables*	*High (Always) n (%)*	*Moderate(Often) n (%)*	*Low (Sometimes)*	*Very low (Rarely) n (%)*	*Mean (SD)*
1. I make arrangement for a skilled birth attendant	54 (37.2)	25 (17.2)	62 (42.9)	4 (2.8)	2.89 (0.951)
2. I make arrangement for a blood donor	23 (15.9)	21 (14.5)	84 (57.9)	17 (11.7)	2.34 (0.885)
3. I make arrangement for someone who will accompany my partner to the hospital	37 (25.5)	45 (31.0)	57 (39.3)	6 (4.1)	2.78 (0.878)
4. I buy things for the arrival of the baby with my spouse	70 (40.3)	63 (43.4)	12 (8.4)	0 (0)	3.40 (0.639)
5. I made arrangement for transportation	58 (40.0)	34 (23.4)	53 (36.6)	0 (0)	3.03 (0.877)
6. I accompany my partner to the hospital myself	56 (38.6)	78 (53.8)	9 (6.2)	2 (1.4)	3.30 (0.647)
7. Keeping money in case of emergency	62 (42.8)	75 (51.7)	8 (5.5)	0 (0)	3.37 (0.589)
8. I stay with my wife during labor and delivery	45 (31.0)	37 (25.5)	59 (40.7)	4 (2.8)	2.85 (0.900)
**Overall mean**					2.99

* Level of involvement: Mean <2.5 low; 2.5–3.25 moderate; >3.25 high.

The level of male involvement in maternity care was moderate, with an overall mean of 2.99 on the 4-point Likert scale. Most respondents indicated participating in maternity care by buying things for the arrival of the baby (3.40), followed by keeping money in case of emergency (3.37) and accompanying their partners to the hospital (3.30), while making arrangement for a blood donor had the lowest mean score (2.34).

On barriers to male involvement in maternity care, their work schedule was reported as the most significant barrier with a mean of 3.58 followed by lack of facilities that involve males in maternity care (3.55) and lack of knowledge on the role of men in maternity care (3.48). However, rejection of assistance by a female partner was reported as the least significant barrier (2.49) (Table 4).

**Table 4:** Barriers to male involvement in maternity care, Nigeria (n=145)

*Variables*	*High (Always) n (%)*	*Moderate(Often) n (%)*	*Low (Sometimes)*	*Very low (Rarely) n (%)*	*Mean (SD)*
1. Financial status	66 (45.5)	65 (44.8)	10 (6.9)	4 (2.8)	3.33 (0.727)
2. Work schedule	85 (58.6)	59 (40.7)	1 (0.7)	0 (0)	3.58 (0.509)
3. Cultural belief	23 (15.9)	102 (70.3)	17 (11.7)	3 (2.1)	3.00 (0.628)
4. Lack of knowledge on the role of men in maternity care	77 (53.1)	64 (44.1)	1 (0.7)	3 (2.1)	3.48 (0.625)
5. Rejection of assistance from female partner	16 (11.0)	52 (35.9)	64 (44.1)	13 (9.0)	2.49 (0.808)
6. Lack of facilities that involve male in maternity care	94 (64.8)	41 (28.3)	6 (4.1)	4 (2.8)	3.55 (0.356)
7. Religious belief	16 (11.0)	58 (40.0)	64 (44.1)	7 (4.8)	2.57 (0.752)
**Overall mean**					3.15

## DISCUSSION

The current study is one of the first to assess male involvement in the south-east region of Nigeria. Knowledge of expected role in maternity care was moderate among the respondents. This may be explained by the high literacy level of the respondents. It is a positive sign, as noted in previous studies, that men who are knowledgeable about maternity care and obtained health education on reproductive health and obstetric matters are more likely to accompany their female partners to antenatal visits^16^.

Moderate knowledge and understanding of their role during maternity care was demonstrated by the respondents in our study. Ignorance was shown to have no association with non-involvement in maternity care in this population since they demonstrated moderate knowledge of their expected role in all the items. To the best of the researchers’ knowledge, no study from a literature search reported good knowledge about maternity care by the respondents^17-19^. The major roles identified by the respondents include helping in taking care of the other children, giving emotional support throughout the pregnancy, reminding the partner of her medications and days for antenatal visits, and helping out with household chores^46^. Our findings agree with those of a study on male involvement during pregnancy and childbirth in rural Ahmadnagar, India, which found that a high percentage of the male partners reported knowledge of their expected roles in assisting in domestic chores, providing food, and emotional support^20^. In contrast, a study in Uganda highlighted that making money available for delivery and postnatal care and ensuring that the woman eats adequately were the perceived roles in maternal health care by male partners, although assisting with household chores was also identified as a role, which corresponds to our findings^21^.

Furthermore, a substantial proportion of the respondents showed moderate level of involvement in maternity care. This is contrary to the prevailing traditional practices in which pregnancy and childbirth have been a female issue. For instance, in the Igbo tradition, the oldest female in the family takes charge of the care of the woman during pregnancy and childbirth while the males are only involved in maternity care after the baby is born. Nevertheless, as also shown by this study, the trend is changing because of the influence of modern civilization and lifestyle on old cultural practices.

The items that the respondents indicated a very high level of participation include: buying things for the arrival of the new baby and spouse; saving money in case of emergency; and accompanying their partner to the hospital and making arrangements for transportation. This finding echoes that of a study that revealed that 84.3% of participants arranged transportation to the hospital for delivery; 62.9% arranged money for delivery^22^, and another that showed that 72.5% of men accompanied their wives to the hospital for their latest delivery^23^. Also, the findings are supported by another study that found a very significant number of male participants accompany their spouses to antenatal care and fully involved during pregnancy and childbirth^20^. In contrast, some studies showed a low level (39.3%) of involvement in accompanying their partners to antenatal visits^22,24^. Also, a local study on the other hand reported that only 32.1% of men in Northern Nigeria ever accompany their partners for maternity care^25^.

Arranging for a blood donor was poorly reported by the respondents, which accounts for a low level of participation in this aspect of maternity care. This may be due to limited emphasis by the midwives on the need for making blood available during delivery or the existing cultural belief or misconception about blood transfusion, which may discourage male partners, or as a result of non-insistence by the hospital management of blood donation as a prerequisite for childbirth.

In this study, lack of facilities that involve the male partner in maternity care, work schedule of the male partner, and lack of knowledge on the role of the male partners during maternity care were the most common barriers identified by the respondents as the reason for low participation in maternity care. Lack of knowledge of the expected role in maternity care as one of the major barriers contradicts earlier findings that revealed good knowledge of the roles of males in maternity care. This contradiction may be attributed to the belief of the respondents that there are some existing roles expected by society and health care workers that are different from their traditional roles. The barrier posed by work schedule could be a result of the nature of their jobs reflected by sociodemographic data that showed a large percentage of the respondents were civil servants. The timing of the clinic and length of time spent in the clinic may discourage them or interfere with the timing of their work. Research evidence also revealed that work considerations^24,26^, financial considerations, negative treatment by heath care workers^27^, women responsibilities^24^, long waiting times and long duration of antenatal care were the major deterrents to male involvement. More so, previous studies^19,26,28^ have shown that the reason for male noninvolvement is the belief that reproductive health issues are exclusively a concern of the women. This was also found to be a predominant belief and posed a significant barrier to male participation in maternity care in this study population with a mean score of 3.0. Rejection of assistance by the female partner was not seen as a barrier to male participation in maternity care. This implies that females in the study locality are willing to accept assistance during pregnancy and childbirth. In contrast, a study carried out in Gambia^27^ reported that men who escorted their partners to clinics were sometimes subjected to gossip by their male counterparts, and interestingly, by women in the clinics who sought antenatal care; consequently this may prompt the pregnant women to discourage their partners in participating to avoid embarrassment. Nevertheless, studies^29-30^ have increasingly shown that women desire their partner’s participation in maternity care. Hence, based on this study, it can be concluded that the level of knowledge of men about maternity care is moderate and their involvement in giving care is moderate. However, peer led and culturally sensitive education and awareness programs should still be carried out by health workers, governmental agencies, nongovernmental organizations and other voluntary groups. This will reinforce what is already known to ensure even more involvement of men in maternal health care. Also, hospital policy reforms and formulation of male-friendly policies that accommodate men in maternal care will be helpful.

### Strengths and limitations

Study strengths include that the respondents were met in their homes regardless of their level of participation in maternity care, this fostered varied responses that yielded robust data. The response rate was relatively high because of convenient timing of data collection. In contrast, limitations include the fact that participants of this study were from a single community with a small sample size, hence our findings may not be generalizable to the broader male population of Nigeria. Also, it is assumed that the respondents may have provided socially desirable answers as the researchers were government officials who they felt may use the information against them.

## CONCLUSIONS

Male involvement is critical for improving maternal and neonatal health indices in Nigeria. This study examined knowledge of the role of males in maternity care, the extent of male involvement and barriers to male involvement in maternity care in Enugu south local government area. The study concluded that male involvement in maternity care is moderate. Although respondents demonstrated moderate knowledge of maternity care, they were restricted by some cultural beliefs. Therefore, men need to be aware of their impact on their spouse’s reproductive health and the need to defy existing cultural norms by involvement in maternity care. Since work schedule was found as a major limitation, the civil service commission should consider granting expectant fathers casual leave for antenatal visits with their partners and paternity leave to be with them during delivery and early puerperium. The hospital administration should also enforce a policy geared towards ensuring that male partners are permitted to share actively in the birth of their child while supporting their partners. This is important and is a major limitation to men who desire to be with their partners.
